# Orbital-dependent Electron-Hole Interaction in Graphene and Associated Multi-Layer Structures

**DOI:** 10.1038/srep17337

**Published:** 2015-11-27

**Authors:** Tianqi Deng, Haibin Su

**Affiliations:** 1 School of Materials Science and Engineering, Nanyang Technological University, 50 Nanyang Avenue, Singapore 639798, Republic of Singapore; 2 Institute of Advanced Studies, Nanyang Technological University, 60 Nanyang View, Singapore 639673, Republic of Singapore; 3 CINTRA, Research Techno Plaza, 50 Nanyang Drive, Singapore 637553, Republic of Singapore

## Abstract

We develop an orbital-dependent potential to describe electron-hole interaction in materials with structural 2D character, i.e. quasi-2D materials. The modulated orbital-dependent potentials are also constructed with non-local screening, multi-layer screening, and finite gap due to the coupling with substrates. We apply the excitonic Hamiltonian in coordinate-space with developed effective electron-hole interacting potentials to compute excitons’ binding strength at M (π band) and Γ (σ band) points in graphene and its associated multi-layer forms. The orbital-dependent potential provides a range-separated property for regulating both long- and short-range interactions. This accounts for the existence of the resonant π exciton in single- and bi-layer graphenes. The remarkable strong electron-hole interaction in σ orbitals plays a decisive role in the existence of σ exciton in graphene stack at room temperature. The interplay between gap-opening and screening from substrates shed a light on the weak dependence of σ exciton binding energy on the thickness of graphene stacks. Moreover, the analysis of non-hydrogenic exciton spectrum in quasi-2D systems clearly demonstrates the remarkable comparable contribution of orbital dependent potential with respect to non-local screening process. The understanding of orbital-dependent potential developed in this work is potentially applicable for a wide range of materials with low dimension.

The optical properties of low-dimensional sp^2^ carbon materials, particularly carbon nanotubes and graphene nanoribbons, are manifested by exciton effects as demonstrated by extensive computational studies including many-body interactions^1^,^2^,^3^,^4^,^5^,^6^, experimental spectroscopic measurements including two-photon excitation^7^,^8^, and reflectance difference techniques^9^. Due to the confinement and weak screening effect, excitons are even observed in the absorption spectrum of metallic carbon nanotubes^10^. The atomic thin layer material, graphene, and its multilayer forms exhibit intriguing features of optical properties associated with the unique electronic structures. For instance, Yang *et al*. found a large excitonic shift at the M point excitation peak by 0.6 eV by solving Bethe-Salpeter equation (BSE) numerically^11^, which was later confirmed in the optical absorption measurements^12^,^13^,^14^. This exciton was interpreted as a resonance with board peak using Fano model^15^, i.e. a discrete state resonating in a background continuum. Yang also proposed a narrow resonant excitonic peak around 12.5 eV arising from the band nesting from parallel π* and σ bands along Γ-K direction^16^. Besides, one narrow and pronounced excitonic peak arising from σ → σ* transitions at 8.3 eV for single-layer and 9.6 eV for bi-layer graphene were computed by Trevisanutto *et al*.^17^. The existence of such a narrow exciton peak was supported by Santoso *et al*. in measuring the optical conductivity of epitaxial multi-layer graphene on SiC with ultraviolet-vacuum ultraviolet reflectrometry and spectroscopic ellipsometry^18^. The pronounced excitonic peak was clearly observed in multi-layer graphenes even at room temperature. However, this excitonic peak position remained almost unchanged for graphene samples with 22 layers or less, in contrast to the clear 1 eV shift of this exciton peak between single- and bi-layer graphene in the BSE results by Trevisanutto *et al*. Thus, despite extensive studies in graphene’s optical properties, the aforementioned controversy invokes more heuristic analysis to gain better understanding of exciton in graphene, particularly by detail account for electron-hole (e-h) interaction in the context of quasi-2D dimension, which represents the 2D nature in structure together with inevitable spreading of wave functions perpendicular to the plane. In this work, to account for both π and σ excitons, we develop the orbital-dependent electron-hole interaction in graphene and its associated multi-layer structures. The unscreened orbital dependent interactions in graphene, with clear quasi-2D character, deviate from Coulomb 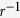 form. We construct non-local screening on the top of Dirac cone approximation^19^, and demonstrate the inclusion of this effect arising from σ and high energy bands to be important for computing exciton binding strength. The thickness dependence of screening in graphene stack with a finite number of layers is also derived by extending the previous work on semi-infinite layered structure^20^. The resulting effective interaction in multi-layer graphene exhibits the explicit orbital- and thickness-dependence. Using these potentials together with ***k∙p*** effective mass method, we compute the binding strength of excitons from π bands at M point and σ bands at Γ point in graphene. Furthermore, we demonstrate the existence of σ exciton in thicker multi-layer graphene stack, and address the coupling between graphene and substrate to be important for clarifying the controversy between the measured little shift in σ exciton peak position of samples on the substrate and computed player-dependence peak shift of σ exciton in the suspended graphene structures. Finally, we perform systematic analysis to scrutinize the contributions of orbital dependent potential and non-local screening to the non-hydrogenic exciton spectrum which appears to be quite general for materials with 2D structures.

## Coordinate-Space Excitonic Hamiltonian with Orbital-dependent Potential

To construct the coordinate-space excitonic Hamiltonian including both effective-mass kinetic term and effective potential, we represent the quasi-particle energy by the sum of k-dependent single-particle kinetic terms and the scissor operator approximation based constant band gap correction. The interaction kernel is approximated by the dominate Coulomb coupling term. Thus, a stationary exciton is the eigenstate of the following BSE in momentum space


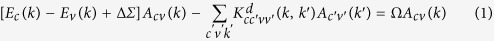


Taking the ***k∙p*** effective mass approximation^21^, the above equation can be transformed into a matrix equation of dimension g=g_c_g_v_, g_c_/g_v_ being the conduction/valence bands degeneracy (See Supplementary Discussion A for details)


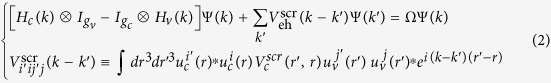


Here ⊗ denotes the Kronecker product, *I*_*g*_ is the identity matrix of order g, and 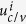 are functions with cellular periodicity. In graphene, for π bands 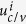 are the cellular parts of Bloch functions, while for σ bands they are chosen to be orthogonal basis functions (See %**Supplementary Discussion B** for details). The Fourier transform of Eq. (2) is


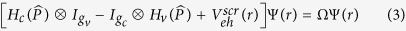


with 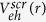 being the Fourier counterpart of 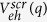. This Schrödinger-type equation describes the equation of motion of an e-h pair interacting via potential 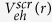
22,23,24. Equations with similar form have also been adopted previously to study multi-layer structures including bi-layer structure of graphene^25^,^26^,^27^, single layer graphene and MoS_2_^28^ with Coulomb type interaction. The Bloch functions 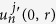 have cellular periodicity and the screened Coulomb interaction can be expanded as 
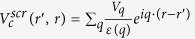
.

So for small k and k′, we have


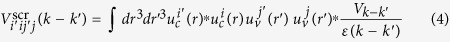


In 3D case, 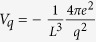 in Planck units (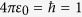) which is adopted throughout this work. So the matrix elements are simple and diagonal, i.e. 
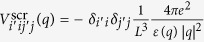
 which means the effective potential is simply the screened Coulomb 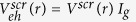. However, in systems like graphene with the distribution of wave function outside the 2D structural plane along z direction considered, we choose the Coulomb term as 
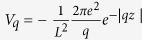
. Then


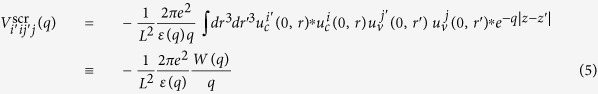


which reminds us of the Coulomb terms between sub-bands in quantum wells 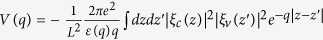 with 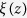 being the sub-band envelope functions^29^.

**Figure 1:**
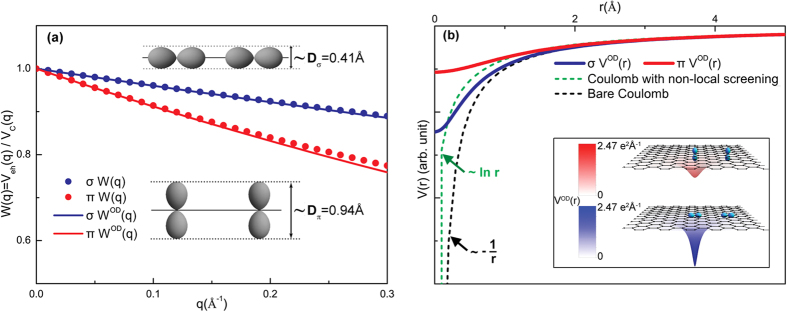
(**a**) Orbital-dependent Coulomb matrix elements for π and σ orbitals. The points indicate the calculated *W*(*q*) from Eq. (6), and the solid lines are the orbital-dependent *W*^OD^(*q*) from Eq. (7). (**b**) A collection of four types of potentials to describe electron-hole interaction. The form of Coulomb interaction with non-local screening is given in ref. 35 by Keldysh, and ref. 36 by Cudazzo, Tokatly and Rubio. The orbital dependent e-h interactions in both π and σ states are shown in inset of Fig. 1b.

To determine*V*_*eh*_, we first evaluate the function *W*(*q*) in matrix elements given by Eq. (5) for σ and π bands in graphene respectively. Considering that the Coulomb matrix elements decay much faster as the momentum transfer *q* increases^30^, we observe that *W*(*q*) can be nicely approximated by an orbital-dependent exponential function *W*^OD^(*q*).


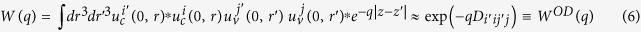


where 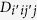 is given in Eq. (7) such that the *W*^OD^(*q*) agrees with the integral *W*(*q*) to the first order of q, which is a good approximation at small q as demonstrated in Fig. 1(a).


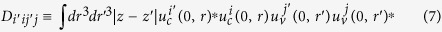


The above Eq. (7) suggests that 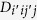 is closely related to the wave functions’ spatial distribution of involving bands along z axis.

If the dielectric function is local and q-independent 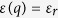, the Fourier transform of *W*^OD^(*q*) is a soft-core Coulomb potential


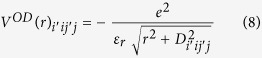


This form coincides with the approximated one observed by Ohno when evaluating two-centre Coulomb integrals between atomic orbitals in Pariser-Parr-Pople method^31^, which is commonly used as Coulomb energy between atomic orbitals^1^,^32^,^33^. Yet, no distinction has been made between π and σ states^33^. It has also been used to describe e-h interaction in graphyne^34^ empirically with the parameter adjusted to fit numerical results. In this work, *V*^OD^ contains the clear physical origin with explicit orbital-dependence beyond the atomic limit, and a well-defined quantity, 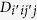, in Eq. (7) rather than a fitting parameter. As plotted in Fig. 1(b), this orbital-dependent potential *V*^OD^ approaches a finite value at small distance, resulting from the finite extension of wave function perpendicular to the 2D atomic plane. It differs from both bare Coulomb between two point charges, which diverges as *r*^−1^ at small distance, and screened Coulomb potential in 2D system which diverges logarithmically^35^,^36^. By Fourier-transforming *V*^OD^ into the coordinate space, we obtained the e-h interaction between σ electrons and between π electrons, as presented in Fig. 1(b). Clearly, the interaction between σ electrons is far stronger than that between π electrons.

The dielectric function has the non-local form 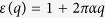 in 2D systems as given by Keldysh^35^ and Cudazzo *et al*.^36^. The key is to develop one orbital-dependent potential with the presence of non-local screening, i.e. 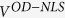, in a similar form of Eq. (8) which can corroborate both short and long range asymptotic limits. Since 
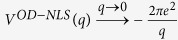
, the long range behavior of this screened potential is same as the bare Coulomb. Around 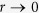, *V*^OD-NLS^ approaches a finite value


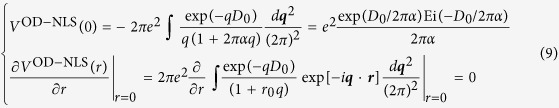



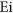
 is the exponential integral. Thus the non-locally screened interaction can be constructed in the form of Eq. (8) with the modified parameter


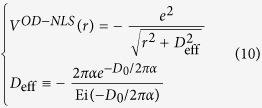


Note that both a larger *D*_*0*_ and stronger non-local polarizability α leads to a weaker e-h potential, esp. at small distance. Hence, the modified orbital-dependent parameter in Eq. (10), *D*_eff_, increases as both *D*_o_ and α increase.

## Screened e-h Interaction in Single-Layer and Multi-Layer Graphene

Consider a perfect 2D system, its static dielectric function is 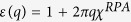 where polarizability 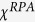 is given by Stern^37^ in random phase approximation (RPA) with g_s_ = 2 being spin degeneracy


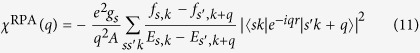


The electronic contributions to 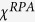 can be partitioned into two categories: those from π states around Dirac points, and those from states further away from Fermi level. The summation over π states in intrinsic graphene has been evaluated^19^


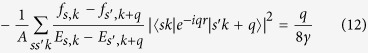


where γ = 6.46 eV · Å is band parameter of graphene^19^. The contributions from other states are also important since they involve σ states arising from carbon (2p_x_, 2p_y_, 2s) orbitals. We notice that in bulk graphite, the states with higher energy difference contribute to the dielectric function in RPA as


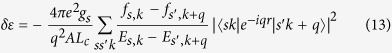


where *L*_*c*_ is the lattice constant of graphite perpendicular to atomic plane. For graphite, the experimental reflection spectrum measurement provided 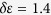 per 2-layer^38^. Assuming 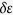 to be similar for graphene and graphite, we combine Eqs (11) and (13) to estimate the contribution from those states, including σ bands, to be 
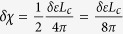
. Hence the static dielectric function in graphene is


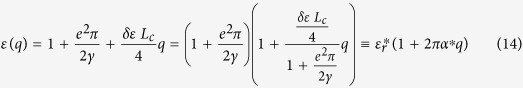


From Eq. (14) we see that π states contribute to the static screening by enhancing the effective relative dielectric constant to 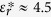, while higher energy states lead to a q-dependent term which yields non-local screening effect similar to 2D insulator^36^. This high energy state term also resembles the non-local dielectric function in semiconducting thin film 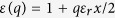 given by Keldysh^35^ with 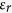 being the bulk relative dielectric constant and *x* being the thin film thickness. In another work by Berkelbach *et al*.^39^, the 2D polarizability of semiconducting transition metal dichalcogenide single layer is also evaluated as 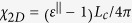 using in-plane dielectric constant 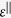 from *ab initio* calculation. These non-local terms arise from the same origin: inter-band polarizability from gapped band pairs. For conduction-valence band pair 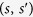 with a finite gap like σ bands in graphene or all bands in semiconductor/insulator, the leading term of matrix element 
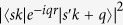
 is the dipole term proportional to *q*^2^, and the energy difference is a finite value. Therefore, the leading term of 2D polarizability given by Eq. (11) is a constant for such band pairs. With this dielectric function as Eq. (14) including the non-local effect from high energy states in graphene, we obtain the electron-hole interaction in graphene using Eq. (10)


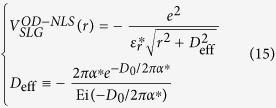


Next, we derive the effective e-h interaction to address the thickness dependence of exciton binding strength in graphene stack. In multi-layer graphene, it is well known that the band structure depends on the stacking sequences of graphene layers. As reported previously^40^,^41^, in intrinsic ABC-stacked N-layer graphene, the density of states diverges when N ≥ 3, hence the screened Coulomb interaction strength converges to zero at small momentum q. In this case, the e-h interaction and excitonic effect vanishes due to the strong screening. Meanwhile in AA-stacked multi-layer graphene, the band structure is similar to a simple stack of decoupled single layer graphene with different Fermi level^42^. Therefore, to emphasize the effect of thickness, we adopt the most common Bernal (AB) stacked multi-layer graphene^43^,^44^,^45^. Since the inter-layer or inter-tube screening plays an important role in e-h interaction in low-dimensional systems^20^,^46^, we extend the work by Visscher and Falicov^20^ on the layer-dependent screening using 2D electron gas model to investigate the thickness effect on the screening in the multi-layer Bernal stacked graphene with band structures. Our system has a stack of N layers separated by interlayer distance d, and each layer has the same polarizability *α*_2*D*_. The Poisson equation for electrostatic potential 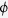 with a point charge in the first layer is


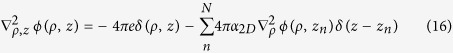


Here *z*_*n*_ = (*n*−1)*d* is the z coordinate of *n*th layer. The solution of Eq. (16) gives the dielectric function for interactions in the surface layer (See %**Supplementary Discussion C** for details)


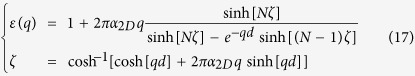


To determine the appropriate α_2*D*_, we first take 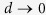, i.e. neglecting the finite thickness of the system, and have 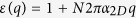. Then we take the average response per layer from Eq. (11) to be the polarizability of each layer,


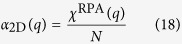


In Eq. (11) the summation indices s/s’ run over all bands. For multi-layer graphene and graphite, we may still simplify Eq. (11) by partitioning these bands into two groups: π bands near K/K′ point, and high energy bands. The contribution from the latter one can be also estimated as 
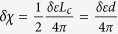
 per layer. By evaluating Eq. (11) for multi-layer with the electronic structure of AB-stacked multi-layer graphene^43^,^44^,^45^ and substituting it into Eq. (18), we obtain the polarizability per layer in multi-layer graphene


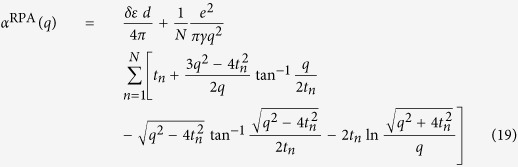


with band parameter *γ* = 6.46 eV · Å, interlayer hopping 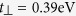 and 
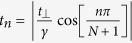
. For multi-layers graphene we obtain the asymptotic form of dielectric function at small q (See %**Supplementary Discussion C** for details)


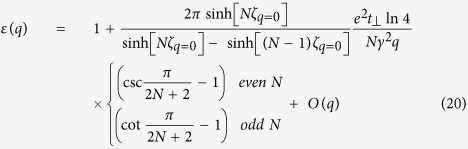


The leading term of dielectric function has Thomas-Fermi screening form 
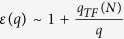
 with thickness dependent Thomas-Fermi wavevector 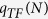. In this case, the orbital-dependent interaction with screening in multi-layer graphene at small q is 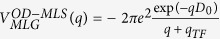. The asymptotic limit at large r (small q) of its Fourier transform is


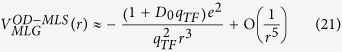


For small r (large q) limit, the exponential term 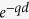 in 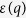 given by Eq. (17) decays fast which yields a negligible inter-layer coupling. Thus, the polarizability from Eq. (19) approaches the value of single layer graphene as shown in its asymptotic form for 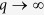, 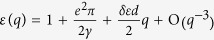. Similar to Eq.(15), such large q behavior yields the following asymptotic form of the effective e-h interaction for small r


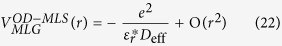


To corroborate the asymptotic behavior at both long- and short-range, we choose the following form to describe the orbital dependent e-h interaction with the presence of multi-layer graphene screening as *ε*_*bg*_


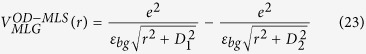


where the parameters 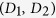 are chosen to subject to both long- and short-range limits in Eqs (21) and (22).

## Results and Discussions

We employ tight-binding wave functions constructed from carbon p orbitals with effective nuclear charge^47^, to compute orbital dependent e-h interaction parameters: 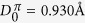 and 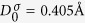. This difference is directly attributed to the distinct spatial distributions of p_z_ and p_x/y_ orbitals along z axis since the wave functions of π bands are combination of p_z_ orbitals while those of σ bands are p_x/y_ orbitals. Interestingly, the effective potential energy of σ exciton is much stronger than that for 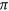 exciton, especially in the small r region, as presented Fig. 1(b). Next, we calculate the band parameters needed in the ***k∙p*** effective mass description of bands in graphene (See %**Supplementary Discussion B** for details) from *ab initio* calculation (See %**Supplementary Method** for details) as 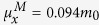, and 
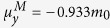
 for π exciton,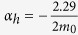,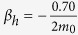, 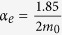 and 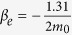 for σ exciton, respectively, where *m*_o_ being the free electron mass. Finally, we solve the excitonic Schrödinger equation in the coordinate space numerically to investigate the characteristics of π exciton at M point and σ exciton at Γ point of graphene and its associated multi-layer forms.

## π Exciton at M Point of Single- and Bi-layer Graphene

**Figure 2:**
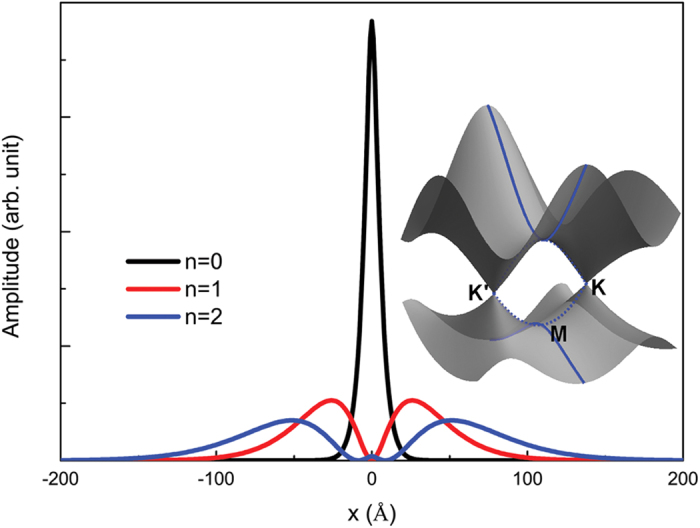
The π exciton’s real-space distribution along positive mass direction at M point in single layer graphene. The amplitude of wave function along positive mass direction x is plotted for excitons with different node number n. The n = 0 exciton have a binding energy of 0.7 eV which is in good agreement with experimental measurements^12^ and GW-BSE calculations^11^. The band structure of graphene is also shown where the saddle point is marked by M, Dirac points are labeled by K and K′.

Since M point is a saddle point, as shown in Fig. 2, the masses have opposite signs. Thus, the solution of Eq. (3) is not a discrete bound state. Considering the fact that the negative mass is much larger than the positive one, we follow Velicky and Sak^48^ and Kane’s^49^ method by separating them into one bound-state related problem and the other scattering-related one,


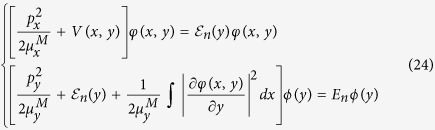


The first equation describes a 1D bound state problem while the second leads to a delocalized scattering state, with a total wave function 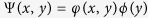. As discussed in the previous section, there is no discrete bound state of saddle point exciton near M point, however, the binding energy of the 1D bound state can be a measure of the Coulomb interaction strength binding energy for this resonant exciton. Using the screened e-h interaction and effective mass of π bands in single layer graphene (SLG), we numerically solve the 1D Schrödinger equation


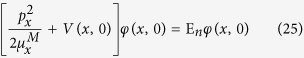


and obtained the binding energy E_*n*_ for first three excited states with n being the number of nodes. The binding energies and parities are shown in Table 1. Considering the symmetry of π bands at M point, only even excitons states are optically active, which means n = 0, 2 are bright while n = 1 is dark. The coordinate-space amplitudes along x axis are plotted in Fig. 2. The exciton with n = 0 has a greater amplitude than that with n = 2. Moreover, the computed binding energy of n = 0 exciton state is 0.73 eV in a good agreement with both experimental value (0.58 eV)^12^ and *ab initio* GW-BSE calculation (0.60 eV)^11^. The slight over-estimation can be attributed to the absent of exchange term in BSE, which would reduce the overall binding strength. For AB-stacked^50^,^51^,^52^ and AA-stacked^53^ bi-layer graphene (BLG), there are four and two inter-π-band transitions respectively, there are multiple electron-hole pairs around M point. However, using the tight binding model with nearest inter- and intra-layer neighbor terms included, we find that the effective mass are the same between the two valence bands (conduction bands), which means the reduced mass of these electron-hole pairs are the same. Meanwhile, only the excitonic shift of one board peak has been reported in both experimental^12^,^14^,^54^ and theoretical^11^,^16^ studies. Therefore, the overall excitonic peak shift can be estimated by solving the 1D bound state in Eq. (25) using one effective screened e-h interaction for BLG. The computed exciton binding energy in BLG is 0.4 eV, which agrees well with experimental result 0.4 eV^12^ and numerical ab initio BSE result 0.45 eV^11^.

The solution of 1D soft-core Coulomb problem has been studied numerically recently by Gebremedhin and Weatherford^55^, where the nodeless ground state (n = 0) has even parity and the eigenvalue approaches negative infinity as the soft-core potential evolves to hard Coulomb potential. So this state is extremely sensitive to the parameter *D* which controls the effective potential. Only when the short range part of the interaction is regularized and no longer diverges as 1/r at small r, the existence of such a nodeless state could be found. For instance, if we only include the non-local screening effect, without the orbital-size regularization, the computed binding energy obtained for SLG is 1.66 eV which is almost three times that of experimental^12^ and GW-BSE calculated^11^ values. Therefore, the existence of such states is one remarkable evidence of the short range regularization of Coulomb interaction. This supports the importance of the orbital-dependent effective potential which naturally arises from the quasi-2D nature of layer materials including graphene.

In this work we mainly focus on the binding strength of exciton, while the absorption intensity by these excitons can be numerically calculated as the imaginary part of frequency dependent dielectric function in the linear response formulism^56^,^57^. It is also possible to simply estimate the absorption intensity using the formula suggested by Elliott^58^ where the matrix element of momentum between ground state and excitonic state is written as the product of inter-band matrix element of momentum and the exciton wave function at 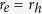. This will shift the oscillator strength of inter-band transitions to an exciton state, which results in an enhanced peak in absorption spectrum^17^,^18^.

## σ Exciton at Γ point of Graphene and Associated Multi-layer Structures

To study σ exciton at Γ point, we start with a variational trial function to find the ground excitonic state for σ bands. Examining the Hamiltonian 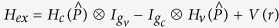, we find that the angular and radial part of eigenfunction can be separated and written as


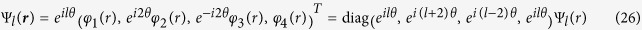


and for each quantum number *l* there is a radial Schrodinger equation


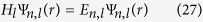


The radial part of normalized 2D hydrogenic orbitals 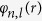 can be used as the variational trial function such that 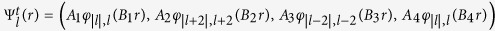, where *A*_*i*_ is the prefactor and *B*_*i*_ is the inverse of effective Bohr radius for each component. For each *l* the energy of the lowest state has an upper bound


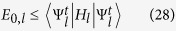


so 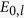 can be approximated with the lower bound of 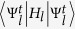. The Hamiltonian leads to degeneracy of states with opposite sign of *l*, and the approximated values of some *E*_0,*l*_ are given in Table 2a. Inspecting the fine structure of energy levels, we note that the binding energies of these bright excitons are only about four times that of *l* *=* *±* *1* and *l* = *±* *3* which have *2p*-like character. Such exciton spectra significantly deviate from standard 2D hydrogenic spectrum^59^ where *1s* state has nine times the energy of *2s*/*2p* state. Such deviation from 2D hydrogenic model has been found in other layered materials in recent years, and will be studied in details in next section. Including the layer-dependent screening effect, we computed the binding energies of excitonic states in multi-layer graphene as shown in Table 2b. The σ exciton has very strong binding energy from 0.51 eV (SLG) to 50 meV (50-layer graphene stack) which is stable against the thermal fluctuation energy even at room temperature (~26 meV) as being observed in the optical conductivity measurement^18^. The stability of σ exciton in thick multi-layer graphene results from the stronger effective e-h interaction of σ state than π state as shown in Fig. 1(b); and the exponential decay in inter-layer screening as distance increases for finite q as discussed in Eq. (17). However, our screening analysis overestimates the screening especially in few-layer graphene on substrates. First, the indirect interlayer coupling lead to the so called trigonal warping in multi-layer graphene^52^,^60^,^61^. This warping effect leads to four massless Dirac cones instead of massive bands, and vanishing density of states around Fermi level. Wang *et al*.^62^ have shown that the dielectric function approaches a finite value ~105 in BLG instead of TF-like 1/q divergence at small q. Secondly, a finite gap opening has been reported in graphene due to strain^63^ and substrate-graphene interaction^64^. According to ARPES measurements of epitaxial few-layer graphene on SiC^64^, finite gaps appear in graphene grown on substrates, due to graphene-substrate interaction. For single-, bi- and tri-layer epitaxial graphene on SiC, the measured gaps are 0.26 eV, 0.14 eV, and 0.066 eV, respectively. To take the finite gap into account, we start from the effective mass model of gapped single-layer graphene^65^ and bi-layer graphene^61^. For intrinsic SLG with a finite gap Δ, the static dielectric function in RPA is obtained by Pyatkovskiy^65^ as


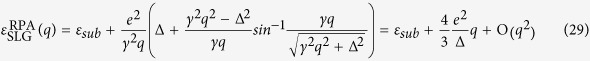


Here *γ* = 6.46 eV · Å is the band parameter of single-layer graphene^19^. Using the effective 2-band Hamiltonian of gapped BLG given by McCann^61^, we obtain the dielectric function for small q,


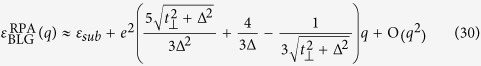


By including the high energy bands effects discussed in Eq. (13) and inter-layer screening effects in Eq. (17), we propose one general dielectric function in gapped SLG and BLG at small q


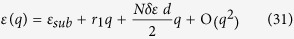


where 
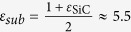
, *r*_1_ ≈ 74 Å for SLG, *r*_1_ ≈ 633 Å for BLG. Since the band structure of AB-stacked tri-layer graphene is equivalent to a bi-layer with the inter-layer hopping parameter 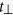 in Eq. (30) replaced by 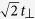, plus a single layer^60^, we characterize the tri-layer graphene (TLG) dielectric function with the above equations of *r*_1_ ≈ 2749 Å. Since the screening parameter *r*_1_ is pretty large here, we need to consider its effect on the long range potential. Considering that *r*_1_ is much greater than *D*_eff_ and 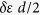, the long range potential is dominated by the screening effect for large r^35^,^36^


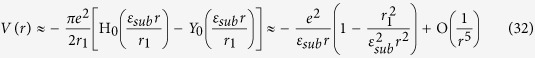


Here H_0_ is the Struve function while *Y*_0_ is Bessel function of the second kind. Since 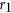 is very large, we will keep both *r*^−1^ and *r*^−3^ terms in approximation. The dielectric function for large q, which is dominated by higher energy dispersions, should be least affected since the gap only affects the low-energy band structure. So, the asymptotic form of e-h interaction for large q is similar to Eq. (22) as 
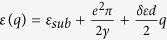
. The asymptotic form at small r will be


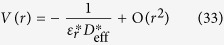


Here 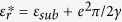, 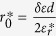 and 
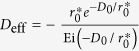
. Subjecting to the asymptotic limits as represented in Eqs (32,33), an effective e-h potential energy form can be written


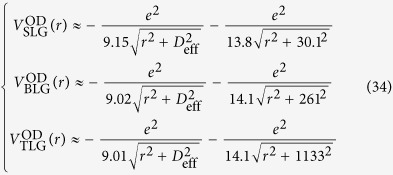


**Figure 3:**
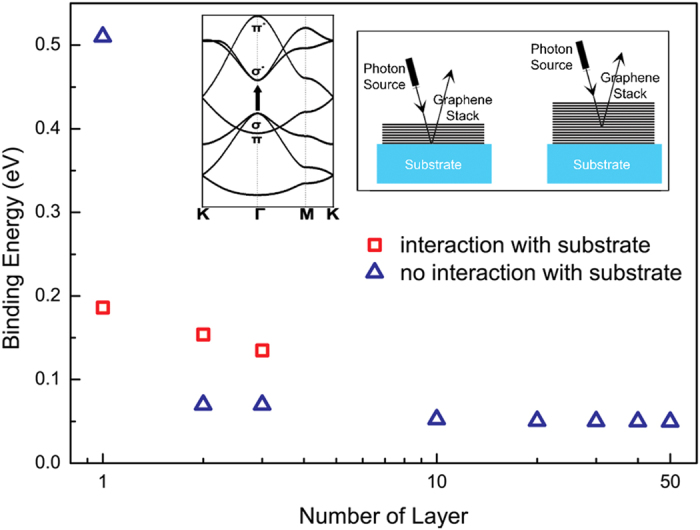
Thickness dependence of σ exciton binding energy in the multi-layer graphene. Red rectangles indicate the binding strength for graphene model on SiC substrate, while blue triangles are for suspended multi-layer graphenes. For graphene stacks on the substrate, the coupling with substrates opens a gap in graphene stacks. The synergized contributions between gap-opening and screening from substrates result in the fairly weak dependence of σ exciton binding energy on the thickness of graphene stacks as shown by data with red square shape. The effect from substrate-graphene interaction weakens as graphene stacks becomes thicker, as illustrated in inset. Thus, even in graphene stack with 50 layers, the σ exciton still has 50 meV binding strength. The existence of σ exciton can be supported by the reflection spectroscopic measurements^18^.

With these effective potentials, the calculated binding energies of σ excitons are 186 meV for SLG, 154 meV for BLG, and 135 meV for TLG, respectively, which are plotted in Fig. 3 together with data in Table 2b. The intrinsic static dielectric response in SLG is weakened by the gap-opening due to the interaction with substrate. The original constant contribution to dielectric function arising from linear bands around Dirac point becomes the non-local screening term *r*_1_*q*, which is quite weak for small q. However, with the enhanced background dielectric constant induced by substrate effect, the total dielectric response at small increases which leads to the reduction of binding energy in SLG as compared to the suspended SLG graphene. Considering the TF like screening in BLG and TLG, the substrate-induced finite gap further regularizes the screening at small q from TF type, which decays as *r*^−3^, to insulator-like for both BLG and TLG. Thus, exciton binding strength is enhanced accordingly. Therefore, since the leading terms of the effective e-h potential in Eq. (32) do not depend on the gap value. the exciton binding energy exhibits a fairly weak dependence on the thickness when the finite gap exists in these structures. Without such gap in multi-layer graphene, there is a finite density of states around Fermi level which lead to the traditional TF screening. Since the band structures of multi-layer graphenes are analogues of single- and bi-layer graphene^60^, we would expect that finite gap will also modify the TF type long-range screening in multi-layer graphene. As the penetration depth of x-ray into HOPG is only 87 Å^66^, around 26 layers at most, the effect of substrate in thicker graphene stacks becomes insignificant on the layers that can be reached by x-ray. And the screening of thicker multer-layer graphenes is similar to that of suspended graphene stacks. For thinner graphene stacks however, substrate-induced gap should be considered. Since the gap size only affect the non-leading term of long range potential, the excitonic peaks in thinner epitaxial graphene shift little as thickness increases. However, one can anticipate that further reduction of exciton binding strength to thermal energy scale at room temperature will lead to the dissociation of exciton, represented by appearance of inter-band transition for the graphene stacks with 75 layers as measured experimentally^18^.

## Non-hydrogenic 2D Exciton Spectrum

As discussed in previous session, a non-hydrogenic feature is identified for σ exciton at M point in graphene by examining the energy gap between *1s* and *2p* excitons with respect to the standard 2D hydrogenic spectrum^59^. Interestingly, for other carbon-based 2D materials, graphane^67^ and graphyne^68^, the exciton with mainly 2p character has a binding energy more than half of 1s exciton’s, and is stronger than that of 2s state, as reported in sp^3^ hydrogenated graphane by Cudazzo *et al*.^36^, and in sp^2^ carbon layer graphyne by Huang *et al*.^34^. In the model proposed by Cudazzo *et al*.^36^, the electron-hole interaction is the Coulomb potential with non-local screening included, while in Huang’s work, the electron-hole interaction has Ohno form with an adjustable fitting parameter.

**Figure 4:**
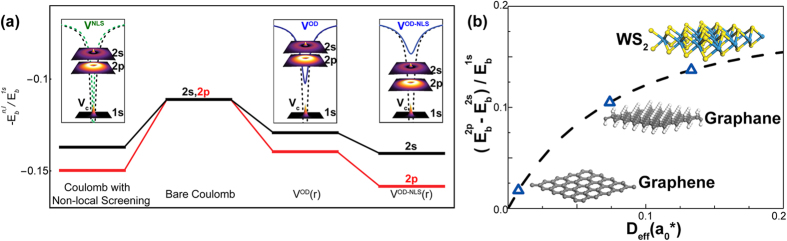
(**a**) 2s-2p exciton splitting (normalized by 1s exciton binding energy) in 2D two-body problems. Solution are calculated using graphene σ band reduced mass together with Coulomb potential, orbital-dependent potential *V*^OD^, non-locally screened Coulomb^35^,^36^ and screened orbital-dependent potential *V*^OD-NLS^, respectively. All energies are normalized by 1s exciton binding energy. Both orbital-size effect and non-local screening weaken the Coulomb interaction at small distance, leading to a range-separated potential which yields the non-hydrogenic exciton spectrum and lifts the 2s-2p degeneracy, as illustrated in the insets. (**b**) Effects of orbital- and screening-dependent parameter, *D*_eff_, on 2s-2p exciton level splitting. Parameter *D*_eff_ is given in effective atomic units. The 2s-2p splitting for graphene, graphane given in ref. 34,36 and WS_2_ given in ref. 69 are illustrated. *D*_eff_ ascends as orbital size and non-local screening increases, which leads to more pronounced deviation from hydrogenic spectrum.

To further understand the role of orbital-size effect and non-local screening, we solve two-body Schrödinger equations using parameters from σ bands. The 2p and 2s excitonic states for such model system, with bare Coulomb potential, *V*^OD^ given Eq. (8), non-locally screened Coulomb potential and *V*^OD-NLS^ given by Eq. (15) are plotted in Fig. 4(a). First, excitonic states with higher principal quantum numbers are closer to the 1s exciton. In the spectra computed by the bare orbital-dependent potential, the ratio between 1s and 2p excitons’ binding energy is around 7.2; and after including non-local screening, this ratio reduces further to 6.3. In 2D hydrogenic model however, this ratio should be 9, and this ratio changes to 6.7 after adding non-local screening to bare Coulomb potential. Furthermore, states with higher angular momenta have lower excitation energies. Instead of 1 in hydrogenic model, the ratios between 2p and 2s excitons’ binding energies (2s-2p splitting) are 1.08 and 1.13 in computations using bare and non-locally screened orbital-dependent potentials; and 1.09 for Coulomb potential with non-local screening, respectively. Such remarkable deviations are the consequences of weakened e-h interaction at small distance. The screening effect, as shown by Cudazzo *et al*.^36^, reduces the *r*^−1^ divergence of Coulomb potential to a logarithmic divergence, and the orbital-size effect further weaken it to a finite value at *r* = 0. From Fig. 4(a) we see that the orbital-size effect and nonlocal screening have comparable contributions to the aforementioned non-hydrogenic features. As illustrated in Fig. 1(b), the orbital-dependent interaction significantly weakens Coulomb interaction at small distance, without adding any dielectric screening. Such weakening is localized and decays as fast as *r*^−3^ at large distance, therefore, very effective on reducing the binding strength of excitons with small principal quantum number n, like *1s* state, due to its tight spatial distribution. And among those states with the same n, states with larger angular quantum number *l* are more diffusive and have less density at small distance. Then the binding energy reduction of, for example, 2s state is greater than the reduction of 2p state, so the 2p state invokes less excitation energy than 2s one. For a given non-local screening, the non-hydrogenic feature becomes more evident as *D* increases since the orbital size information is nicely included in *D*. (See %**Supplementary Fig. S2** for details). Thus, it is interesting to compare the exciton 2s-2p splitting observed in WS_2_ and graphane. With two-photon luminescence measurement and GW-BSE calculation Ye *et al*.^69^ found that the s-series and p-series of excitons in WS_2_ significantly deviate from 2D hydrogenic model. By matching the ratio between 2s-2p splitting and 1s binding energy with our model, we observed that WS_2_ has greater 2s-2p splitting than graphane as shown in Fig. 4(b). Using reduced mass and polarizability given in Ref. 39 by Berkelbach, Hybertsen and Reichman, together with Eq. (15), we obtain an orbital-dependent parameter 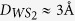 which is comparable with d-orbital spatial extension in WS_2_. This also supports the important role of orbital-size dependence. Finally, we address the possibility of observing the 2s-2p splitting in graphene. The strong screening of π state imposes the formidable challenge in forming spectroscopically resolved series π exciton states. Motivated by the desirably stable σ exciton in graphene stacks, we estimate 2s-2p splitting of σ excitons in a suspended graphene to be in the order of 10 meV.

## Conclusions

In summary, we construct an orbital-dependent effective potential describing e-h interaction in graphene to unravel its quasi-2D characteristics originated in the orbitals’ spatial distribution. The modified e-h potentials are also developed by including effects of non-local screening, multi-layer screening, and the coupling with substrates. We demonstrate the inclusion of orbital dependence to be crucial for correctly accounting for the binding energy of π exciton at M point. We highlight the stronger e-h interaction in σ orbitals than that of π ones, which facilitates the formation of stable σ exciton in graphene stacks even at room temperature. The coupling with substrates opens a gap in graphene stacks. The synergized contributions between gap-opening and screening from substrates result in the fairly weak dependence of σ exciton binding energy on the thickness of graphene stacks. The orbital dependent potential plays a comparable role as non-local screening in scrutinizing the non-hydrogenic exciton spectrumin in quasi-2D materials.

## Additional Information

**How to cite this article**: Deng, T. and Su, H. Orbital-dependent Electron-Hole Interaction in Graphene and Associated Multi-Layer Structures. *Sci. Rep*. **5**, 17337; doi: 10.1038/srep17337 (2015).

## Supplementary Material

Supplementary Information

## Figures and Tables

**Table 1:** Binding energies of first three 1D bound states at M point in single layer graphene.

	Exciton binding energy in SLG (eV)	Parity of exciton wave function
n = 0	0.73	Even
n = 1	0.062	Odd
n = 2	0.035	Even

The experimental measurements^12^ and GW-BSE calculations^11^ suggested exciton’s binding strength of 0.58 eV and 0.60 eV, respectively.

**Table 2:**
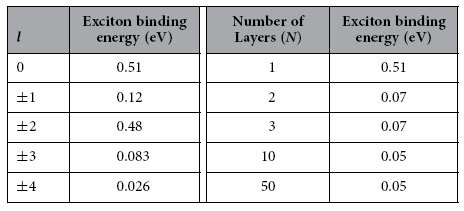
a. Left: Quantum number *l*-dependent binding energy for σ exciton at Γ point in single layer graphene. b. Right: Number of layers (*N*) dependent binding energy for lowest σ exciton at Γ point in suspended multi-layer graphene stack.
