# Supplementary material for: Orbital-dependent Electron-Hole Interaction in Graphene and Associated Multi-Layer Structures
Source: Sci Rep. 2015 Nov 27;5:17337. doi: 10.1038/srep17337 (PMC4661528; doi:10.1038/srep17337)
Supplement: Supplementary Information [file srep17337-s1.doc]

Orbital-dependent Electron-Hole Interaction in Graphene and Associated Multi-Layer Structures

Tianqi Deng^1,2,3^ and Haibin Su^1,2,3,*^

^1)^ School of Materials Science and Engineering, Nanyang Technological University, 50 Nanyang Avenue, Singapore 639798, Republic of Singapore

^2)^Institute of Advanced Studies, Nanyang Technological University, 60 Nanyang View, Singapore 639673, Republic of Singapore

^3)^CINTRA, Research Techno Plaza, 50 Nanyang Drive, Singapore 637553, Republic of Singapore

*Email: hbsu@ntu.edu.sg

# Supplementary Discussion A

# Effective Mass Excitonic Hamiltonian

With **k∙p** effective mass approximation[1], one can expand the cellular part of a Bloch wave function at small **k** as

$$u_{n}\left(k\right)=\sum_{n'} C_{nn'}\left(k\right)u_{n'}\left(0\right)$$

In the case when contributions from other bands are neglected due to symmetry or large energy difference, only those bands in the same subspace are kept. Thus, we have

$$\psi_{nj}\left(k,r\right)=e^{ikr}\sum_{j'}^{g} C_{nj}^{j'}\left(k\right)u_{n}^{j'}\left(0,r\right)$$

Then the single-particle Hamiltonian $H_{0}$ and eigenfunction $\psi_{nj}=u^{T}P\left(k\right)e^{ik\cdot r}$ are matrices which satisfy

$$H_{0}\left(k\right)P\left(k\right)=P\left(k\right)\Lambda\left(k\right)$$

with

$$\begin{matrix} u=\left[u_{n}^{1}\left(0,r\right),u_{n}^{2}\left(0,r\right),\cdots,u_{n}^{g}\left(0,r\right)\right]^{T} \\ P\left(k\right)=\left[\begin{matrix} C_{n1}^{1}\left(k\right) & \cdots& C_{ng}^{1}\left(k\right) \\ \vdots& \ddots& \vdots\\ C_{n1}^{g}\left(k\right) & \cdots& C_{ng}^{g}\left(k\right) \end{matrix}\right] \\ \Lambda\left(k\right)=\text{diag}\left(E_{n1}\left(k\right),E_{n2}\left(k\right),\cdots,E_{ng}\left(k\right)\right) \end{matrix}$$

Assuming conduction/valence bands have degeneracy of g_c_/g_v_, Eq. (1) in main text becomes

with $V^{scr}$ being the screened Coulomb interaction. For simplicity we employ a new notation, $|t\rangle=|cv\rangle$, with dimension g=g_c_g_v_ such that

$$A\left(k\right)=\text{vec}\left(\left[\begin{matrix} A_{c_{1}v_{1}}\left(k\right) & \cdots& A_{c_{1}v_{g_{v}}}\left(k\right) \\ \vdots& \ddots& \vdots\\ A_{c_{g_{c}}v_{1}}\left(k\right) & \cdots& A_{c_{g_{c}}v_{g_{v}}}\left(k\right) \end{matrix}\right]\right)=\left[A_{t_{1}}\left(k\right),A_{t_{1}}\left(k\right),\cdots,A_{t_{g}}\left(k\right)\right]^{T}$$

Then Eq. reduces to the matrix form

$$\begin{matrix} \left[\Lambda_{c}\left(k\right)\otimes I_{g_{v}}-I_{g_{c}}\otimes\Lambda_{v}\left(k\right)+\delta\Sigma\right] A\left(k\right)+ \\ \sum_{k'} P_{eh}\left(k\right)^{\dagger}V_{eh}^{\text{scr}}\left(k-k'\right)P_{eh}\left(k'\right)A\left(k'\right)=\Omega A\left(k\right) \end{matrix}$$

Here $P_{eh}=P_{c}\otimes P_{v}$,denotes the Kronecker product[2], and$I_{g}$is the identity matrix of order$g$. Based on the properties of Kronecker product, we have the following relation

$$\left[P_{c}\otimes P_{v}\right]^{\dagger}\left[H_{c}\otimes I_{g_{v}}-I_{g_{c}}\otimes H_{v}\right]\left[P_{c}\otimes P_{v}\right]=\Lambda_{c}\otimes I_{g_{v}}-I_{g_{c}}\otimes\Lambda_{v}$$

With this Eq. becomes

$$\begin{matrix} \left[H_{c}\left(k\right)\otimes I_{g_{v}}-I_{g_{c}}\otimes H_{v}\left(k\right)\right]P_{eh}\left(k\right) A\left(k\right)+ \\ \sum_{k'} V_{eh}^{\text{scr}}\left(k-k'\right)P_{eh}\left(k'\right)A\left(k'\right)=\Omega P_{eh}\left(k\right)A\left(k\right) \end{matrix}$$

which describes an eigenvalue problem on the wave function $\Psi\left(k\right)=P_{eh}\left(k\right) A\left(k\right)$.

# Supplementary Discussion B

# Band Structure and k∙p Hamiltonian of Graphene

The band structure of graphene σ and π bands has been studied using linear combination of atomic orbitals (LCAO) by Painter and Ellis [3]. And Saito, Dresselhaus and Dresselhaus [4] have successfully reproduced the band structure using tight-binding model with carbon 2s and 2p orbitals. With tight-binding parameters reported in Ref [4], the tight-binding band structure of graphene is plotted in Fig. S1 as follows:


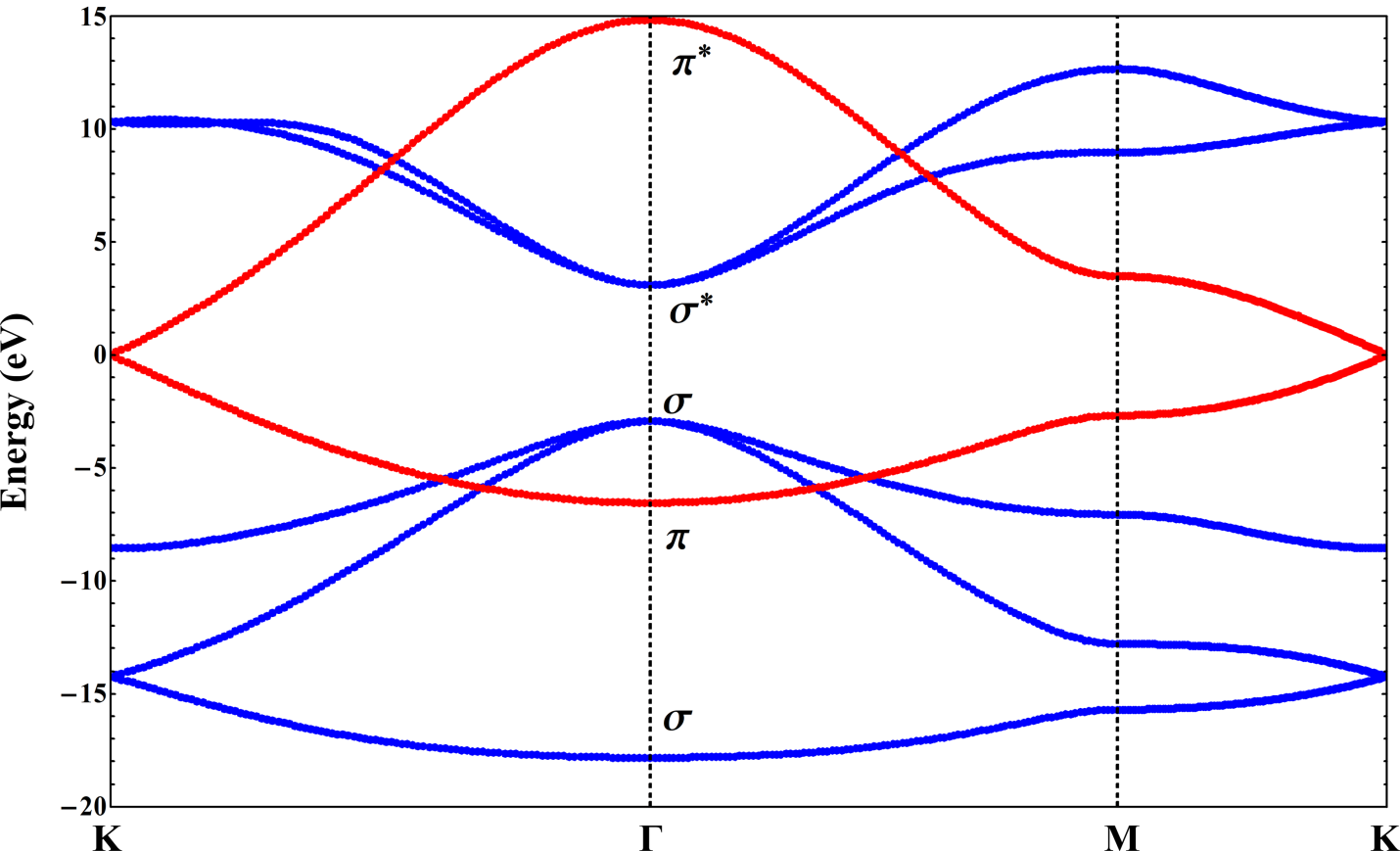


Figure S1. Tight-binding band structure of π and σ bands in graphene using parameters developed by Saito, Dresselhaus and Dresselhaus [4].

From the tight-binding model, wave functions can be obtained for each k point. Considering the parabolic shapes of σ bands around Γ point and π bands around M point, we can employ a second-order ***k∙p*** effective mass model given in Appendix A to capture the main features of these bands.

By solving the tight-binding model we found that the highest σ and lowest σ* bands at Γ point are both doubly degenerate and their wave functions at Γ point where k=0 are simple linear combination of p_x_/p_y_ orbitals on two carbon sites, say$\psi_{\sigma}^{x/y}\left(k=0,r\right)=\Phi_{x/y}^{A}+\Phi_{x/y}^{B}$. The other two σ bands are further away from Fermi level and are linear combinations of carbon 2s orbitals $\Phi_{2s}^{A}\pm\Phi_{2s}^{B}$. Therefore within the ***k∙p*** approximation, the effective mass Hamiltonians are in the matrix form. And the Bloch functions around Γ point can be constructed as combination of $\psi_{\sigma*/\sigma}^{x/y}\left(k=0,r\right)$. The non-mixing among s and p_x_/p_y_ can be also understood using the group theory analysis by Kogan, Nazarov, Silkin and Kaveh [5]. Given the symmetry group of these bands, E_2g_ and E_1u_ of D_6h_ group respectively, we use the method of invariance[1] and obtain the single particle effective mass Hamiltonian for electrons and holes in σ bands as

$$\begin{matrix} H_{0}^{h}\left(p\right)=\alpha_{h}I_{2}\left(p_{x}^{2}+p_{y}^{2}\right)+\beta_{h}\left[\sigma_{3}\left(p_{x}^{2}-p_{y}^{2}\right)-\sigma_{1}2p_{x}p_{y}\right] \\ H_{0}^{e}\left(p\right)=\alpha_{e}I_{2}\left(p_{x}^{2}+p_{y}^{2}\right)+\beta_{e}\left[\sigma_{3}\left(p_{x}^{2}-p_{y}^{2}\right)+\sigma_{1}2p_{x}p_{y}\right] \end{matrix}$$

with$I_{2}=\left[\begin{matrix} 1 & 0 \\ 0 & 1 \end{matrix}\right]$,$\sigma_{1}=\left[\begin{matrix} 0 & 1 \\ 1 & 0 \end{matrix}\right]$ and $\sigma_{3}=\left[\begin{matrix} 1 & 0 \\ 0 & -1 \end{matrix}\right]$. The corresponding eigenvalues are $E_{h}^{\pm}=\left(\alpha_{h}\pm\beta_{h}\right)k^{2}$ and $E_{e}^{\pm}=\left(\alpha_{e}\pm\beta_{e}\right)k^{2}$ so these parameters can be obtained from *ab initio* band structure. The corresponding wave functions are written as linear combinations of $\psi_{\sigma*/\sigma}^{x/y}\left(k=0,r\right)$. To simplify, we may instead use complex orbital and change the basis to$u_{h}^{\pm}\left(r\right)=\Phi_{\pm}^{A}-\Phi_{\pm}^{B}$, $u_{e}^{\pm}\left(r\right)=\Phi_{\pm}^{A}+\Phi_{\pm}^{B}$ with$\Phi_{\pm}=\Phi_{x}\pm i\Phi_{y}$. Then the Hamiltonians become

$$\left\{\begin{matrix} H_{0}^{h}\left(p\right)=\left[\begin{matrix} \alpha_{h}I_{2}\left(p_{x}^{2}+p_{y}^{2}\right) & \beta_{h}\left(p_{x}+ip_{y}\right)^{2} \\ \beta_{h}\left(p_{x}-ip_{y}\right)^{2} & \alpha_{h}I_{2}\left(p_{x}^{2}+p_{y}^{2}\right) \end{matrix}\right] \\ H_{0}^{e}\left(p\right)=\left[\begin{matrix} \alpha_{e}I_{2}\left(p_{x}^{2}+p_{y}^{2}\right) & \beta_{e}\left(p_{x}+ip_{y}\right)^{2} \\ \beta_{e}\left(p_{x}-ip_{y}\right)^{2} & \alpha_{e}I_{2}\left(p_{x}^{2}+p_{y}^{2}\right) \end{matrix}\right] \end{matrix}\right.$$

The π bands at M point is non-degenerate so the effective mass Hamiltonian is

$$H_{0}^{M}\left(p\right)=\frac{p_{x}^{2}}{2\mu_{x}^{M}}+\frac{p_{y}^{2}}{2\mu_{y}^{M}}$$

where $\mu_{x/y}^{M}$ are effective masses to be obtained from simple LDA-DFT band structure.

# Supplementary Discussion C

# Dielectric function in layered structures

The Poisson equation for electrostatic potential with a point charge in the first layer is

Here $z_{n}=\left(n-1\right)d$ is the z coordinate of *n*th layer. The 3D Fourier transform is

The 2D Fourier component in each layer can be defined as an inverse Fourier transform, so a Fourier transform of Eq. is

Solving this equation we obtain the total potential generated in the first layer

The Eq. gives the dielectric function for interactions in the first layer which has the following form

$$\varepsilon\left(q\right)=1+2\pi\alpha_{2D}q\frac{\sinh\left[N\zeta\right]}{\sinh\left[N\zeta\right]-e^{-qd}\sinh\left[\left(N-1\right)\zeta\right]}$$

To determine the appropriate$\alpha_{2D}$, we take the limit$\text{ d}\to0$, say neglecting the finite thickness of the system, and arrive at $\varepsilon\left(q\right)=1+N2\pi\alpha_{2D}q$. Then we average take the average response per layer from Eq. (11) in main text as the polarizability of each layer, arriving at

$$\alpha_{2\text{D}}\left(q\right)=\frac{\chi^{\text{RPA}}\left(q\right)}{N}$$

In Eq. (11) from main text the summation indices s/s’ run over all bands. For multi-layer graphene and graphite, we may still simplify it by partitioning these bands into two groups: π bands near K/K’ point, and high energy bands. The contribution from the latter one is also estimated as$\delta\chi=\frac{1}{2}\frac{\delta\varepsilon L_{c}}{4\pi}=\frac{\delta\varepsilon d}{4\pi}$ per layer. By evaluating Eq. (11) for multi-layer with the electronic structure of AB-stacked multi-layer graphene[6-8] and substituting it into Eq. we obtain the polarizability per layer in intrinsic N-layer graphene

$$\begin{matrix} \alpha^{\text{RPA}}\left(q\right)=\frac{\delta\varepsilon d}{4\pi}+\frac{1}{N}\frac{e^{2}}{\pi\gamma q^{2}}\sum_{n=1}^{N} \left[t_{n}+\frac{3q^{2}-4t_{n}^{2}}{2q}\tan^{-1}\frac{q}{2t_{n}}\right. \\ \left.-\sqrt{q^{2}-4t_{n}^{2}}\tan^{-1}\frac{\sqrt{q^{2}-4t_{n}^{2}}}{2t_{n}}-2t_{n}\ln\frac{\sqrt{q^{2}+4t_{n}^{2}}}{q}\right] \end{matrix}$$

with $\gamma=6.46\text{eV}\cdotÅ$, $t_{\perp}=0.39\mathrm{eV}$and $t_{n}=\left|\frac{t_{\perp}}{\gamma}\cos\left[\frac{n\pi}{N+1}\right]\right|$. Taking the limit $t_{n}\to0,N\to1$ we recover the result of single layer graphene given in Eq. (14) from main text. For multi-layers however, it is quite complicated. To evaluate the dielectric function in Eq. , we need to evaluate $\zeta$ first. Examining the behavior of $\alpha_{2D}$ we see that for$q<<\frac{t_{\perp}}{\gamma}$,

According to Eq. we have

It approaches a finite constant at small q and substituting Eqs. and into Eq. we obtain the asymptotic form of dielectric function at small q as

# Supplementary Method

# *ab initio* Calculation of Effective Mass Parameters.

To obtain the effective mass parameters in our model, we carried out calculation using Vienna *ab initio* Simulation Package (VASP)[9-12] within generalized gradient approximation (GGA) (Perdew-Burke-Ernzerhof exchange correlation functional)[13]. The ion–electron interaction was modeled by the projector augmented wave (PAW) method[14,15]. The graphene unit-cell has lattice parameter $a=0.246nm$ and an inter-layer spacing of 2nm to eliminate the undesirable individual layers coupling due to the periodicity of the supercell. 65 irreducible k-points are employed for self-consistent calculation and band dispersion along Γ-M and M-K path are calculated using a finer mesh of 41 points along the path. The effective masses are evaluated as the second derivatives of band energy with respect to k.

# Supplementary Figure 2S


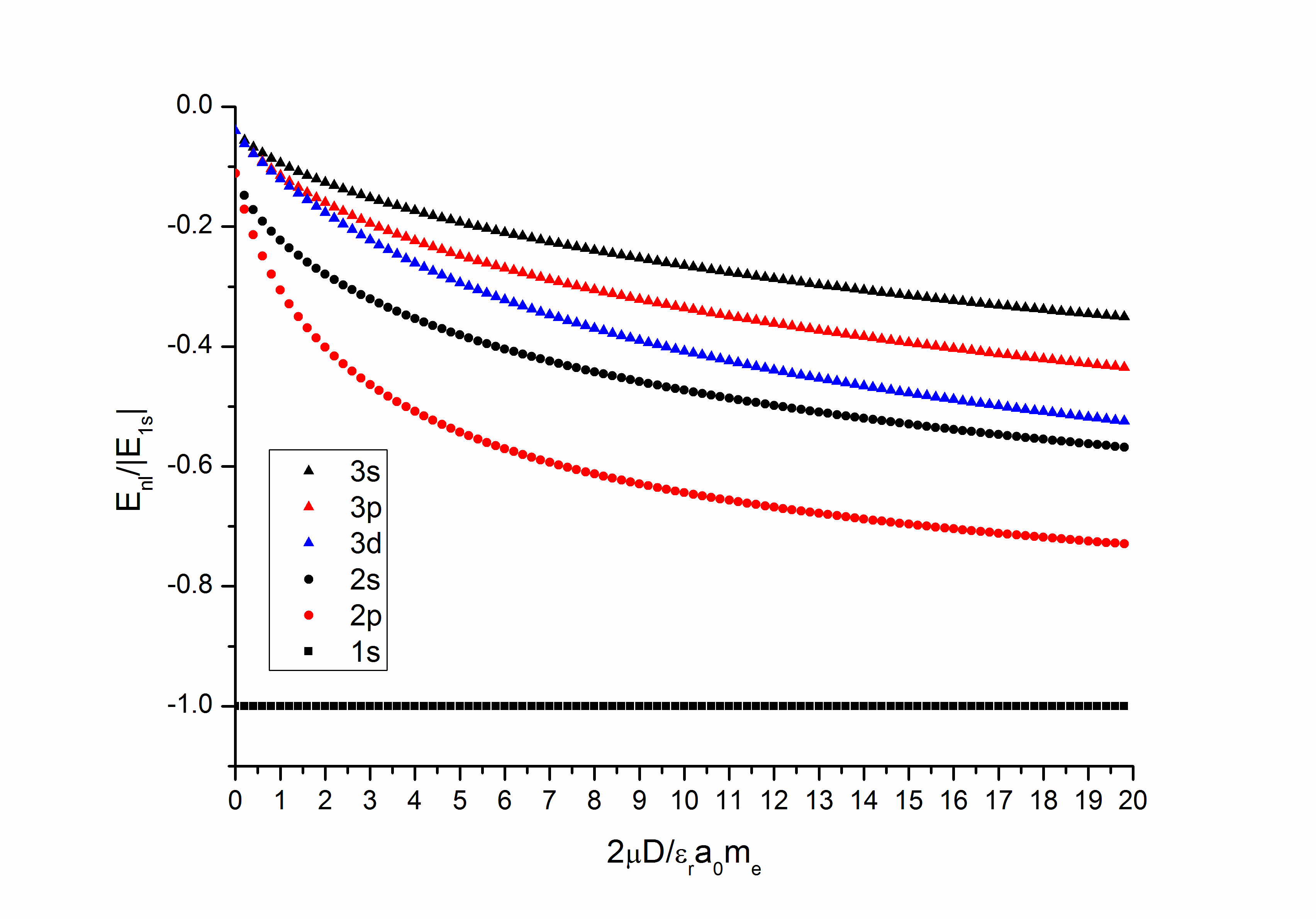


Figure S2 Parameter D dependence of non-hydrogenic exciton spectrum. As D increase which means orbital size becomes larger or non-local screening becomes stronger, the deviation from hydrogenic model is more evident.

The orbital-dependent interaction induces a significant weakening of Coulomb interaction at small distance. Such weakening is localized and decays as fast as$r^{-3}$at larger distance. Therefore, such weakening of potential energy is more effective on excitons with smaller principal quantum number n, like 1s state, which is much more localized, and the binding energies for states with smaller n are reduced more significantly. And among those states with the same n, states with larger angular quantum number l are more diffusive and have vanishing density at small distance. Then the binding energy reduction of, for example, 2s state is greater than the reduction of 2p state, so the degeneracy is lifted. The deviation and splitting becomes greater as the parameter *D* increases. Since *D* is determined by the wave function spatial distribution and non-local polarizability, we anticipate the non-hydrogenic deviation to be more significant in quasi-2D materials with bigger orbitals and stronger non-local screening.

**References:**

[1] L. C. Lew Yan Voon and M. Willatzen, *The k-p method : electronic properties of semiconductors* (Springer, Dordrecht ; New York, 2009).

[2] A. J. Laub, *Matrix analysis for scientists and engineers* (Society for Industrial and Applied Mathematics, Philadelphia, 2005).

[3] G. S. Painter and D. E. Ellis, *Phys. Rev. B* **1**, 4747 (1970).

[4] R. Saito, G. Dresselhaus, and M. S. Dresselhaus, (World Scientific.

[5] E. Kogan, V. U. Nazarov, V. M. Silkin, and M. Kaveh, *Phys. Rev. B* **89**, 165430 (2014).

[6] T. Ando and M. Koshino, *ELECTRONIC STATES OF GRAPHENE AND ITS MULTI-LAYERS* (World Scientific Publ Co Pte Ltd, Singapore, 2009), Proceedings of the 9th International Symposium on Foundations of Quantum Mechanics in the Light of New Technology.

[7] H. K. Min and A. H. MacDonald, *Prog Theor Phys Supp*, 227 (2008).

[8] F. Guinea, A. H. Castro Neto, and N. M. R. Peres, *Phys. Rev. B* **73**, 245426 (2006).

[9] G. Kresse and J. Furthmüller, *Phys. Rev. B* **54**, 11169 (1996).

[10] G. Kresse, *J Non-Cryst Solids* **193**, 222 (1995).

[11] G. Kresse and J. Hafner, *Phys. Rev. B* **49**, 14251 (1994).

[12] G. Kresse and J. Hafner, *Phys. Rev. B* **47**, 558 (1993).

[13] J. P. Perdew, K. Burke, and M. Ernzerhof, *Physical Review Letters* **77**, 3865 (1996).

[14] G. Kresse and D. Joubert, *Phys. Rev. B* **59**, 1758 (1999).

[15] P. E. Blöchl, *Phys. Rev. B* **50**, 17953 (1994).
